# Levetiracetam Modulates EEG Microstates in Temporal Lobe Epilepsy

**DOI:** 10.1007/s10548-022-00911-2

**Published:** 2022-09-13

**Authors:** Lorenzo Ricci, Pierpaolo Croce, Patrizia Pulitano, Marilisa Boscarino, Filippo Zappasodi, Flavia Narducci, Jacopo Lanzone, Biagio Sancetta, Oriano Mecarelli, Vincenzo Di Lazzaro, Mario Tombini, Giovanni Assenza

**Affiliations:** 1 Unit of Neurology, Neurophysiology, Neurobiology, Department of Medicine, University Campus Bio-Medico of Rome, via Álvaro del Portillo, 21, 00128 Rome, Italy; 2 Department of Neuroscience, Imaging and Clinical Sciences, G. d’Annunzio University of Chieti-Pescara, Chieti, Italy; 3 Department of Human Neurosciences, Policlinico Umberto I, Sapienza University of Rome, Rome, Italy; 4 Institute for Advanced Biomedical Technologies (ITAB), G. d’Annunzio University of Chieti-Pescara, Chieti, Italy; 5 Neurorehabilitation Department, IRCCS Salvatore Maugeri Foundation, Institute of Milan, Milan, Italy

**Keywords:** Temporal lobe epilepsy, EEG, Biomarkers, Levetiracetam, Microstates

## Abstract

To determine the effects of Levetiracetam (LEV) therapy using EEG microstates analysis in a population of newly diagnosed Temporal Lobe Epilepsy (TLE) patients. We hypothesized that the impact of LEV therapy on the electrical activity of the brain can be globally explored using EEG microstates. Twenty-seven patients with TLE were examined. We performed resting-state microstate EEG analysis and compared microstate metrics between the EEG performed at baseline (EEG_pre_) and after 3 months of LEV therapy (EEG_post_). The microstates A, B, C and D emerged as the most stable. LEV induced a reduction of microstate B and D mean duration and occurrence per second (*p* < 0.01). Additionally, LEV treatment increased the directional predominance of microstate A to C and microstate B to D (*p* = 0.01). LEV treatment induces a modulation of resting-state EEG microstates in newly diagnosed TLE patients. Microstates analysis has the potential to identify a neurophysiological indicator of LEV therapeutic activity. This study of EEG microstates in people with epilepsy opens an interesting path to identify potential LEV activity biomarkers that may involve increased neuronal inhibition of the epileptic network.

## Introduction

Temporal Lobe Epilepsy (TLE) is a common neurological disorder and the most frequent cause of focal epilepsy in adults (Engel et al. 2012). About 70% of people with epilepsy achieve seizure freedom with anti-seizure medications (ASMs) (Brodie et al. 2012), while 30% will develop drug-resistant epilepsy defined as “failure of adequate trials of two tolerated and appropriately chosen and used ASMs schedules (whether as monotherapies or in combination) to achieve sustained seizure freedom” (Kwan et al. 2010). ASM treatment failure may occur for several reasons, including the inability to reach the brain or because of insufficient pharmacological response (Premoli et al. 2017). In order to understand the reason for treatment failure in epilepsy, the measurement of the modifications induced by ASMs in the human brain is of paramount importance (Premoli et al. 2017; Ricci et al. 2021).

For people with TLE, the electroencephalogram (EEG) is a pivotal neurophysiological technique in both guiding clinical management and supporting diagnosis (Koutroumanidis et al. 2017). The measurement of quantitative EEG parameters to evaluate the effect of specific drugs on the electrical activity of the brain is known as pharmaco-EEG (Jobert et al. 2012). Pharmaco-EEG has already shown promising results in measuring the effects of psychiatric medications (Mucci et al. 2006; Iosifescu 2011), and as an established tool for the classification of new drugs (Fink 2010). Indeed, pharmaco-EEG has numerous advantages as an analytic tool in that it can provide a multidimensional approach for the evaluation of brain activity by assessing the dynamics of several features at the same time (i.e., frequency power, connectivity and complexity analysis) (Tong and Thankor 2009) and its use in epilepsy research is still far to be fully exploited (Höller et al. 2018). Pharmaco-EEG analysis applied to epilepsy has the potential to effectively predict therapeutic efficacy (Croce et al. 2021) and to objectively measure the neurotoxicity of ASMs (Saletu et al. 1986).

Yet, the use of the EEG to investigate the brain activity at rest is a nontrivial task since the signal of interest is of low amplitude and it may be difficult to characterize the underlying neural sources (Custo et al. 2017). To address this challenge, numerous previous works have used the principles of electric field topographical analysis and showed that resting-state EEG could be represented as a sequence of scalp topographies, the so-called “microstates”, those configurations remain semi-stable for short time periods of about 40–100 ms (Lehmann et al. 1987; Michel and Koenig 2018). These scalp potential topographies derive from the synchronous activation of various cortical areas reflecting different functions (Lehmann et al. 1987). As such, microstates are able to offer a global topographical representation of specific neural processes without any kind of a-priori hypotheses, providing a promising analytic approach for resting-state EEG analysis (Khanna et al. 2015; Ricci et al. 2020).

Along this line, the goal of this study is to measure the effects of one of the most prescribed ASM, Levetiracetam (LEV) (Nicholas et al. 2012), in a population of newly diagnosed TLE using resting-state EEG microstate analysis. We hypothesize that the impact of a first ASM therapy on the electrical activity of the brain in TLE can be globally explored using EEG microstates, that could eventually represent potential neurophysiological biomarkers of LEV activity and efficacy. To test our hypothesis, we performed a resting-state EEG microstate analysis on a population of TLE people and compared microstate features between the EEG performed before LEV initiation (EEG_pre_) and the EEG performed 3 months after LEV therapy (EEG_post_).

## Materials and Methods

### Subjects and Data Collection

The research team retrospectively reviewed data from newly diagnosed TLE patients enrolled at the epilepsy clinic of Department of Human Neurosciences of Policlinico Umberto I University Hospital of Rome and of Campus Bio-Medico University of Rome between January 2016 and January 2021. The data have been previously used for other studies from our group and selection criteria for patients in our cohort can be found elsewhere (Croce et al. 2021). The study protocol received approval by the ethics committee of Policlinico Umberto I Ethic Board-Rome- and Campus Biomedico University Ethic Board-Rome.

### EEG Recording

All patients underwent registration with nineteen channel-EEG according to the international 10/20 system (Micromed, Mogliano Veneto, IT). The reference was placed on FPz and the ground on FCz. Impedance was kept below 5 kOhm for all electrodes. A sampling rate of 256 Hz was used for these recordings. The resting EEG recording lasted 15 min and was performed with closed eyes, with patients seated in a comfortable armchair in a quiet room (Croce et al. 2021).

### Microstates Analysis

**Fig. 1:**
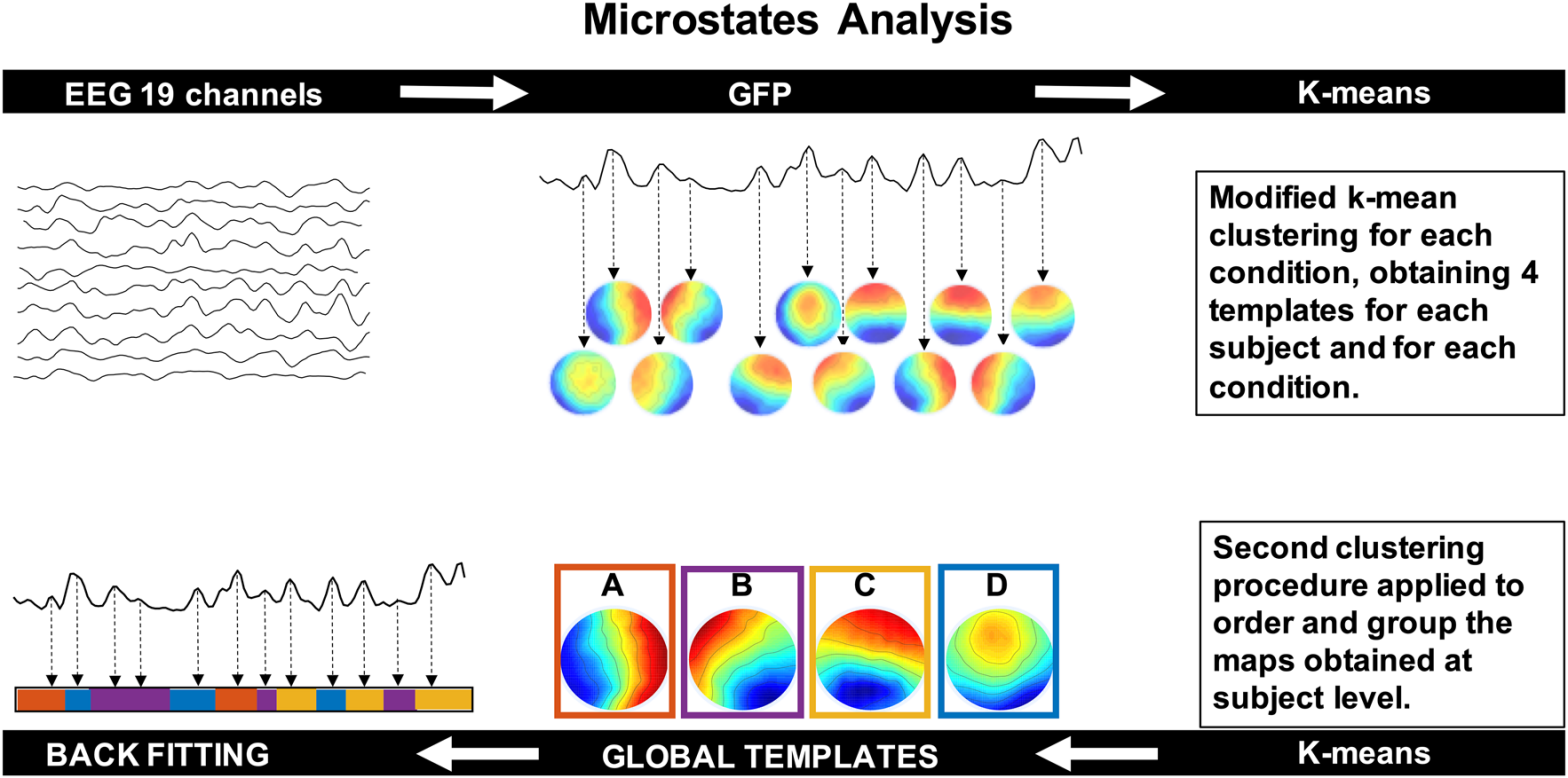
Microstate analysis pipeline. Step 1: The intervals of stable topographical configurations are identified. Step 2: The global templates of the dominant microstates are calculated for the identified intervals of brain functional stability. Step 3: The identified global templates are backfitted to each noise-free EEG dataset to find the specific sequence of microstates on which metrics are calculated

With microstates analysis, it is possible to depict the ongoing brain dynamics by reducing the EEG time course to a fixed number of dominant topographical configurations or global templates (i.e., microstates). Once identified the global templates, it is possible to calculate quantitative metrics that describe the sequence of microstates. Typical metrics are mean duration, coverage, occurrence and probability of transition of each microstate. The pipeline of microstates analysis is summarized in Fig. 1. Data processing was performed following the OHBM COBIDAS MEEG good practice recommendations (Pernet et al. 2020).

#### Identification of Moments of Stable Topographical Configurations

We calculated the EEG Global Field Power (GFP) for each subject and each condition (i.e., EEG_pre_ and EEG_post_). GFP is given by the standard deviation of the EEG signal amplitude across all electrodes at a given time instant and is a reference-free descriptor of the potential field strength. GFP peaks are considered to correspond to intervals of highest topographical stability (Murray et al. 2008). Thus, the scalp topographies corresponding to GFP peaks were retained for the subsequent steps necessary to identify the microstate templates (Fig. 1).

#### Global Templates Extraction

To identify the global templates representative of the dominant microstates for a condition, two clustering operations were applied sequentially. The first clustering procedure was applied to individual EEG datasets to identify the optimal number of microstate templates, i.e., the number of microstate templates that explain most of the variance of the EEG signals in the individual datasets. The second clustering procedure was applied to all sets of individual microstate templates to identify, using a spatial correlation algorithm, the global microstate templates. This procedure was applied to EEG recordings from each condition: EEG_pre_ and EEG_post_. Since the clustering procedure is a supervised algorithm, the optimal number of clusters (microstates templates) needs to be estimated. To this aim, we applied a clustering k-algorithm, varying k from 2 to 12. The optimal number of k was identified by applying the Krzanowski-Lai (KL) criterion: optimal k was chosen as the k corresponding to the second KL maximum value (Murray et al. 2008).

Checking if conditions-wise templates (EEG_pre_/EEG_post_) are similar between groups is a required step to compute global microstates templates. The topographical similarity between template pairs was assessed by means of the topographical analysis of variance (TANOVA) (Brunet et al. 2011; Wagner 2019). Such analysis is based on the evaluation of effect size between conditions. The effect size is quantified by computing the global dissimilarity (GD) between pairs of global microstate templates as:

$${GD}_{u,v}= \sqrt{\frac{1}{N}\sum_{i=1}^{N}(\frac{{u}_{i}}{{GFP}_{u}}-\frac{{v}_{i}}{{GFP}_{v}}{)}^{2}}$$

where ${u}_{i}$ and ${v}_{i}$ are the electric potentials of the i-th electrode in the microstate templates u and v respectively; ${GFP}_{u}$ and ${GFP}_{v}$ are the global field powers of the microstate templates (u and v); N is the number of electrodes (hence of electric potential values in each microstate template). ${GD}_{u,v}$ GDu,v is indirectly related to the spatial correlation between two maps. Indeed, the lower the global dissimilarity, the higher the spatial correlation. With this procedure, two sets of k global microstate templates were obtained, one for each condition of EEG recordings (EEG_pre_/EEG_post_).

#### Backfitting of the Global Templates and Microstates Metrics Calculation

For each subject, the global microstate templates were backfitted to the EEG signals of each condition (EEG_pre_/EEG_post_), by calculating the spatial correlation between each global template and the scalp potential distributions at each GFP peak. A winner-take-all procedure was applied to assign at each time interval centered to the GFP peak the microstate with the highest spatial correlation. With this procedure, the EEG time courses of each condition were reduced to a sequence of microstates. From this sequence, the following metrics were calculated (Lehmann et al. 1987): (i) mean microstate duration (ms); (ii) mean microstate occurrence per second (Hz); (iii) mean percentage of covered analysis time (%); and (iv) directional predominance between microstates (%). The directional predominance between two global microstate templates X and Y was defined as the difference between the probability of transition from X to Y and the probability of transition from Y to X. A positive value of directional predominance indicates that the probability of transit from X to Y is higher than the probability of transit from Y to X (the opposite for a negative value) (Lehmann et al. 2005).

### Statistical Analysis

Differences in the microstates’ global template explained variance between condition (EEG_pre_/EEG_post)_ were assessed by paired t-test. The differences of microstate metrics (microstate duration, occurrences per second, percentage of covered analysis time) among conditions (EEG_pre_/EEG_post_) were evaluated by aligned rank transform (ART) ANOVA for non-parametric repeated-measures designs (Wobbrock et al. 2011), with *Condition* (two levels: EEG_pre_, EEG_post_), *Template* (levels equal to the number of microstate templates) and *Side* (two levels: right TLE [r-TLE] and left TLE [l-TLE]) as within-subject factors. In this method, an ANOVA on ranks is calculated for each of these factors after aligning the data by subtracting the effect of the other factors. Significant main effect of *Condition* was followed up by Bonferroni corrected post-hoc comparisons using the ART-C algorithm for multifactor contrast tests in R (Elkin et al. 2021), to compare microstate metrics across the templates.

To assess differences in directional predominance between conditions, for each transition probability an ART ANOVA design was applied, with *Condition* (two levels: EEG_pre_, EEG_post_), *Pairs* (levels equal to the number of the possible template pairs) and *Side* (two levels: r-TLE and l-TLE) as within-subject factor. Post-hoc comparisons were Bonferroni corrected. Clinical outcome (seizure-free vs. non seizure-free) and the presence of a structural abnormality as aetiology for epilepsy (structural vs. non-structural) were used as covariates in the ANOVA models. Seizure freedom was defined as the absence of seizures or auras for at least 2 years on unchanged medications (Stephen and Brodie 2002) based on patient self-reporting and clinical diary. Patients’ clinical characteristics were compared between seizure-free and non seizure-free patients using the χ^2^ test. Significance level was set at *p* < 0.05. Results are reported as mean ± standard deviation unless differently stated. The statistical analysis was performed using the R statistical packages (Team 2013).

## Results

### Patients Clinical Characteristics

Twenty-seven patients with TLE (15 females) satisfying all the selection criteria were included in the study (Table 1). Fourteen patients (51.9%) presented a r-TLE, whereas 13 patients (48.1%) presented l-TLE. Sixteen patients (59.3%) achieved seizure-freedom after the introduction of LEV, eight patients (29.6%) presented a > 50% reduction in seizure frequency, whereas three patients (11.1%) presented a < 50% reduction in seizure frequency after LEV. The mean age at the time of the TLE diagnosis was 48.4 ± 22.5 years (range: 20–86 years). Eleven patients (40.7%) presented an abnormal MRI as a cause of their epilepsy, with different diagnoses (Table 1). Five patients (18.5%) experienced non-serious adverse events related to LEV therapy. The mean LEV maintenance daily dose after approximately three months was 1222.2 ± 381.88 mg (range: 750–2000 mg).

**Table 1:** Clinical features of our cohor

N	Sex	Age, (ys)	Seizure frequency	Semiology	Aetiology	EEG focus	Outcome	LEV maintenance dose (mg)	Adverse events
1	F	73	Yearly	FS with PA	Structural (Ischaemic Stroke)	Left TP	NSF (SR < 50%)	1000	No
2	M	36	Yearly	FTB	Structural (Ischaemic Stroke)	Right FT	NSF (SR > 50%)	1000	No
3	M	64	Monthly	FS with IA	Unknown	Right T	NSF (SR < 50%)	1000	Transient depressive symptoms
4	F	25	Yearly	FS with IA	Structural (Cavernous Malformation)	Right T	NSF (SR > 50%)	1000	No
5	F	20	Monthly	FS with IA/FTB	Unknown	Right T	NSF (SR > 50%)	1000	Irritability
6	M	69	Monthly	FS with IA	Unknown	Left T	NSF (SR > 50%)	2000	No
7	F	30	Yearly	FS with IA	Unknown	Right FT	NSF (SR > 50%)	2000	No
8	F	38	Monthly	FS with IA	Unknown	Right T	NSF (SR > 50%)	1000	No
9	F	69	Monthly	FS with IA/FTB	Structural/Infectious (History of HSV Encephalitis)	Left T	NSF (SR < 50%)	1000	Transient depressive symptoms
10	M	64	Yearly	FS with IA/FTB	Structural/Infectious (Cerebral Abscess)	Left T	NSF (SR > 50%)	1000	No
11	F	24	Monthly	FTB	Structural (Hippocampal Sclerosis)	Left T	NSF (SR > 50%)	1500	No
12	M	28	Monthly	FTB	Unknown	Right T	SF	1500	No
13	F	55	One episode	FS with IA/FTB	Unknown	Left T	SF	2000	No
14	M	26	Monthly	FTB	Unknown	Right FT	SF	1500	No
15	M	78	Five episodes	FS with IA	Unknown	Left T	SF	1000	No
16	F	58	One episode	FS with PA	Unknown	Right T	SF	1000	Drowsiness
17	M	47	Two episodes	FS with IA/FTB	Structural (Cerebral AVM)	Left T	SF	2000	No
18	F	77	Four episodes	FS with PA	Structural (Ischaemic Stroke)	Left T	SF	1000	No
19	M	75	One episode	FS with PA	Unknown	Left T	SF	1000	Nausea
20	F	24	Four episodes	FS with IA	Structural (Cavernous Malformation)	Left T	SF	1250	No
21	F	63	One episode	FS with IA	Structural (Cavernous Malformation)	Right T	SF	1000	No
22	M	20	Monthly	FTB	Unknown	Left T	SF	1000	No
23	F	86	Monthly	FS with PA	Structural (Meningioma)	Left TP	SF	1000	No
24	M	39	Yearly	FTB	Unknown	Right T	SF	1500	No
25	M	75	Yearly	FTB	Unknown	Right T	SF	1000	No
26	F	21	Yearly	FS with IA/FTB	Unknown	Left T	SF	750	No
27	F	24	Yearly	FTB	Unknown	Right T	SF	1000	No

*Ys* years, *LEV* Levetiracetam, *M* male, *F* Female, *FS* Focal seizures, *FTB* focal to bilateral tonic–clonic seizures, *PA* Preserved awareness, *IA* Impaired awareness, *T* Temporal, *FT* Fronto-Temporal, *TP* Temporo-Parietal, *SF* Seizure-Free after 2 years of therapy, *NSF* Non-Seizure Free, *SR* Seizure Reduction, *AVM* Arteriovenous malformation, *HSV* Herpes Simplex Virus

### Optimal Number of Microstates

**Fig. 2:**
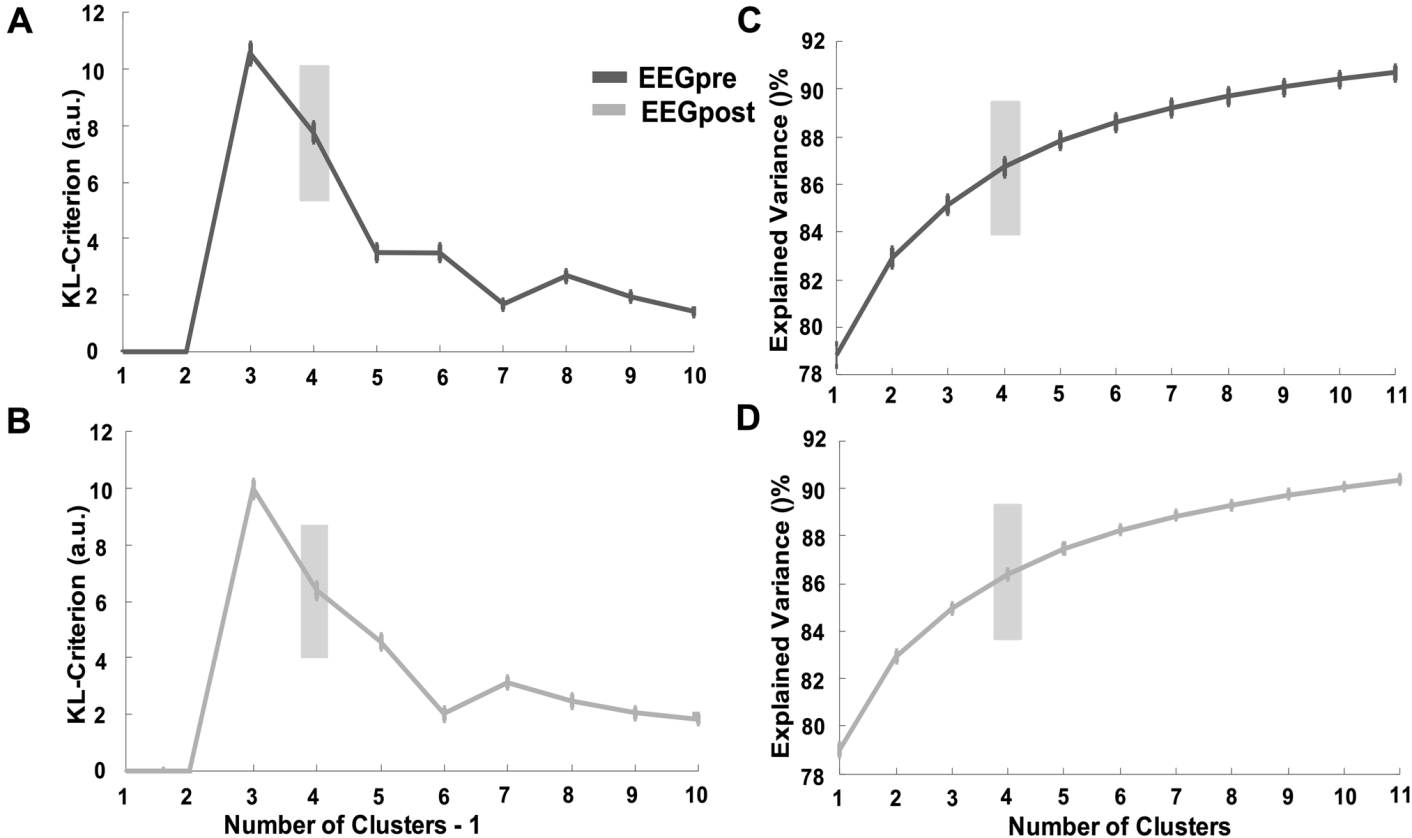
Microstate optimal templates’ number. **A** and **B** KL criterion trend with respect to the number of clusters. The second maximum is 4 for both EEGpre and EEGpost conditions. **C** and **D** Global explained variance as a function of the number of clusters. There is no significant increase from four and up clusters. EEGpre: EEG performed before the initiation of Levetiracetam (LEV) therapy. EEGpost: EEG performed after 3 months of LEV therapy

**Fig. 3:**
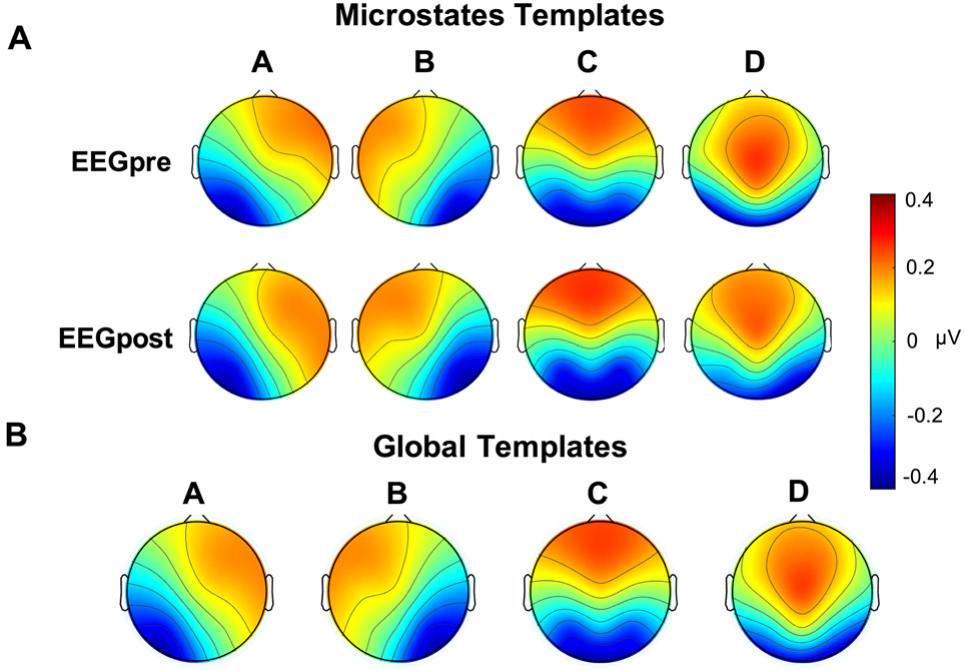
Microstates (A–D) template for each condition. **A** EEGpre: EEG performed before the initiation of Levetiracetam (LEV) therapy. EEGpost: EEG performed after 3 months of LEV therapy. **B** Microstate maps (from A to D) represent the global microstate templates obtained from condition-wise microstate template

Applying the KL criteria for optimal number of microstates, we identified four templates for both conditions (EEG_pre_/EEG_post_). Figure 2a, b shows the KL criterion for both EEG_pre_ and EEG_post_ conditions. TANOVA analysis revealed no difference between the templates from the two conditions (*p* > 0.05). For this reason, global templates were calculated as described in the methods section. According to the topographies of microstates obtained in previous works, the templates were labeled as A, B, C and D (Fig. 3a). The global templates were then used for the backfitting procedure (Fig. 3b).

### Explained Variance

The explained variance for the template extracted was 86.1 ± 0.2% and 86.2 ± 0.5% in the EEG_pre_ and in the EEG_post_ condition, respectively (Fig. 2c, d). We found no significant differences in the explained variance between conditions (*p* > 0.05).

### Microstates Metrics

**Fig. 4:**
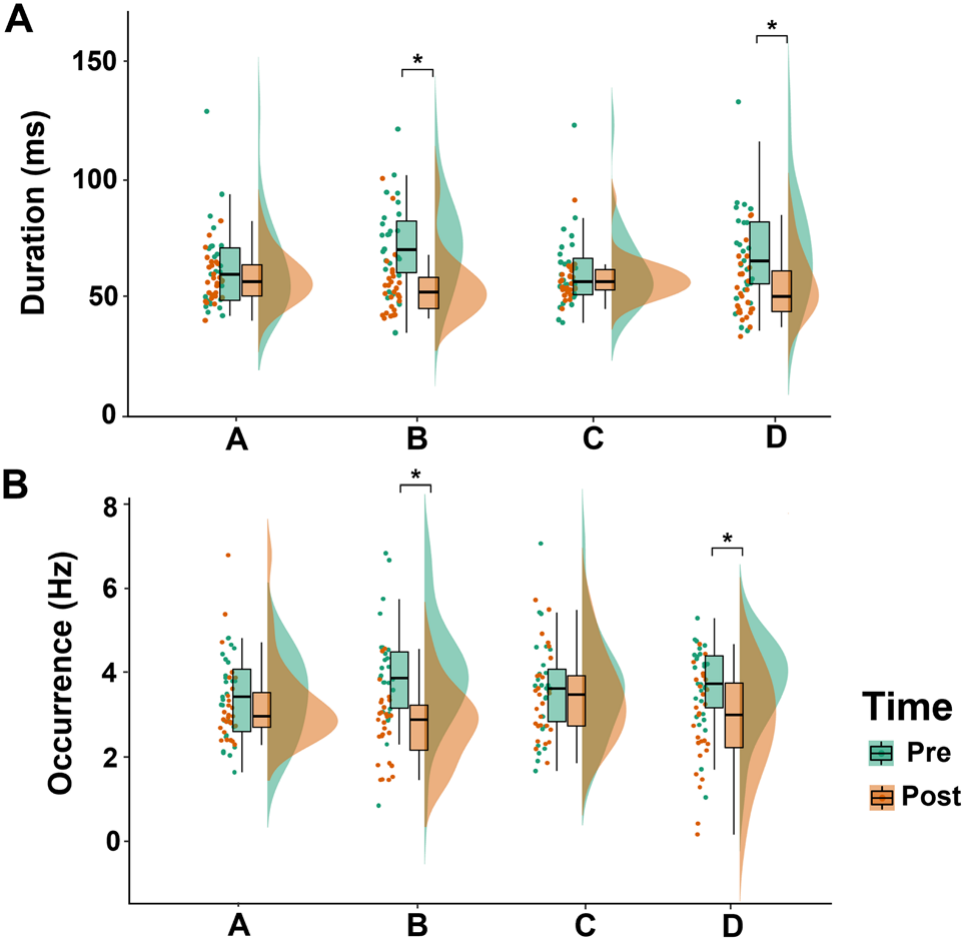
Microstate metrics across conditions. Raincloud plot and boxplot distribution of microstate mean duration (**A**) and occurrences per second (**B**) comparing the EEG performed before Levetiracetam (LEV) initiation (Pre) and the EEG performed after 3 months of LEV therapy (Post) across different microstate templates. Black lines represent mean values. Circles denote mean metrics value for each subject. We found a global reduction of microstate metrics in the Post condition (*p* < 0.05). Post-hoc tests revealed a specific reduction in microstate B and D mean duration and occurrences per second after 3 months of LEV therapy. *Bonferroni corrected *p* < 0.05

The ART ANOVA showed a significant main factor Condition for both mean duration (F_(1, 208)_ = 22.89, *p* < 0.001; Fig. 4a) and occurrences per second (F_(1, 208)_ = 20.72, *p* < 0.001; Fig. 4b). Both metrics were lower in the EEGpost condition than in the EEGpre condition (Fig. 4).

A significant *Condition* x *Template* interaction was also found (F_(3, 208)_ = 5.59, *p* = 0.001 for mean duration; F_(3, 208)_ = 3.17, *p* = 0.02 for occurrences per second). Post-hoc comparisons revealed that the mean duration of microstate B and D were shorter (*p* < 0.01) in the EEG_post_ condition (Fig. 4a). Similarly, the occurrences per second of microstates B and D were lower (*p* < 0.01) in the EEG_post_ condition (Fig. 4b).

We found no significant differences in microstate metrics after controlling for clinical outcome (F_(1, 200)_ = 2.18, *p* = 0.14 for mean duration; F_(1, 200)_ = 0.34, *p* = 0.56 for occurrences per second) and for structural aetiology of TLE (F_(1, 200)_ = 1.18, *p* = 0.28 for mean duration; F_(1, 200)_ = 3.21, *p* = 0.07 for occurrences per second).

### Directional Predominance

**Fig. 5:**
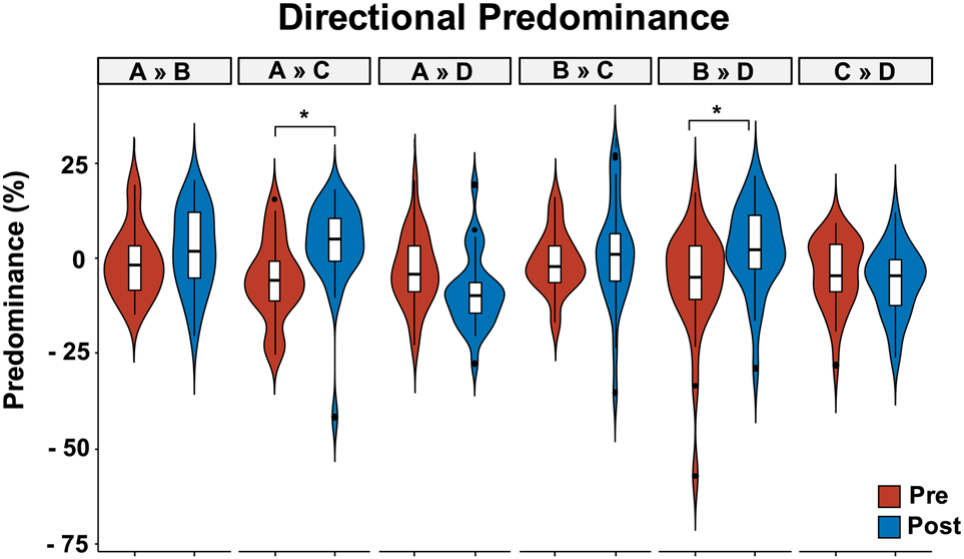
Directional Predominance. Boxplot and violin plot distributions of the directional predominance of the four microstate templates across conditions (Pre vs. Post). Circles denote values that are farther than 1.5 interquartile ranges. Pre EEG performed before the initiation of Levetiracetam (LEV) therapy. Post EEG performed after 3 months of LEV therapy

The ART ANOVA with *Condition* and *Pairs* as within-subject factors showed a global modification of the directional predominance in the EEG_post_ condition (F_(1, 312)_ = 6.03, *p* = 0.01, Fig. 5). A significant *Condition* x *Pairs* interaction was also found (F_(5, 312)_ = 5.38, *p* < 0.001). Post-hoc comparison revealed that the A to C and B to D directional predominance were increased (*p* < 0.01 and *p* = 0.04, respectively) in the EEG_post_ condition (Fig. 5).

We found no significant differences in the directional predominance after controlling for clinical outcome (F_(1, 300)_ = 0.14, *p* = 0.71) and for structural aetiology of TLE (F_(1, 300)_ = 0.18, *p* = 0.44).

### Microstate Results According to Temporal Lobe Epilepsy Side

**Fig. 6:**
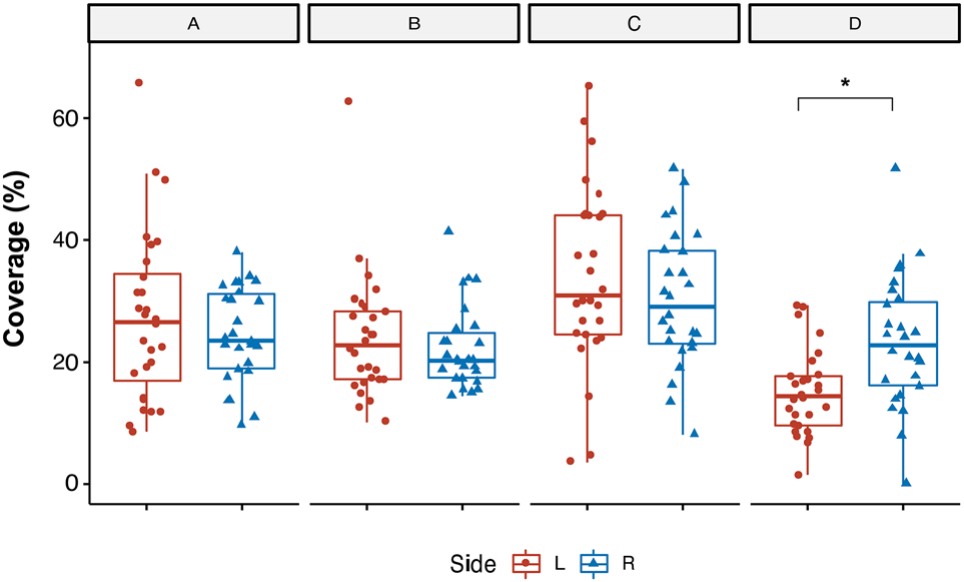
Microstate metrics modifications comparing right side (R) and left side (L) Temporal Lobe Epilepsy (TLE). Boxplot distributions of mean percentage of covered analysis time among patients with right-TLE (R, in blue) and left-TLE (L, in red). Circles and triangles indicate mean microstate values for each subject considering both EEGpre (before Levetiracetam) and EEGpost (after Levetiracetam) conditions. We found lower microstate D coverage values in l-TLE as opposed to r-TLE patients. *Bonferroni corrected *p* < 0.05

**Fig. 7:**
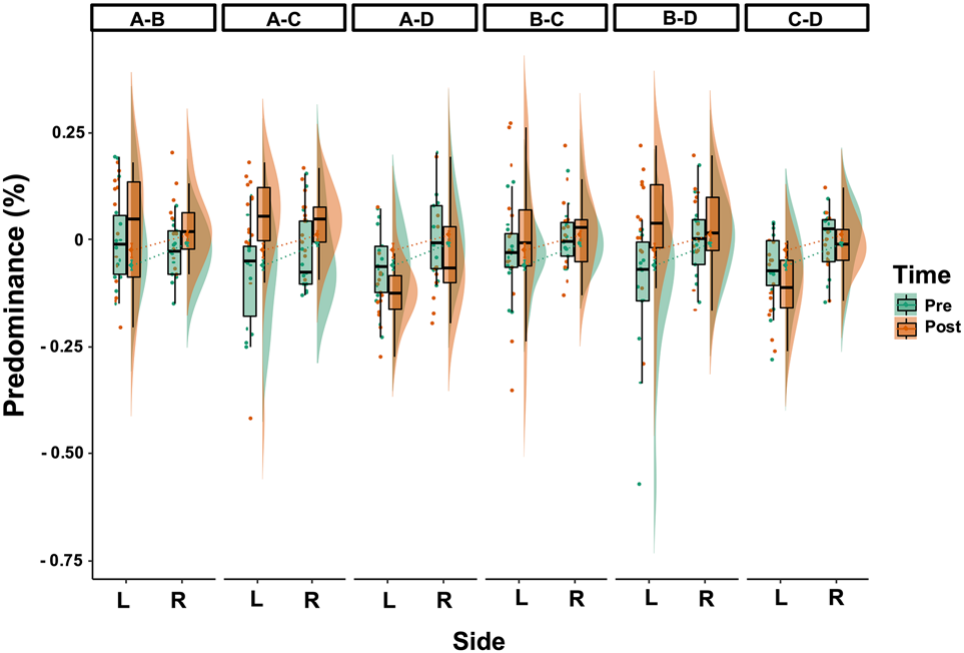
Directional predominance comparing right side (R) and left side (L) Temporal Lobe Epilepsy (TLE). Raincloud plot and boxplot distribution of microstate directional predominance comparing patients with right-TLE (R) and left-TLE (L) and the EEG performed before Levetiracetam (LEV) initiation (Pre) and the EEG performed after 3 months of LEV therapy (Post) among different microstate templates. Black lines represent mean values. Circles denote mean metrics value for each subject. We found reduced values of microstates’ directional predominance in left-TLE as opposed to right-TLE across all microstate pairs (*p* < 0.05)

We observed that the side of TLE had an effect on specific microstate metrics. In particular, a significant *Template x Side* interaction was found for the percentage of covered analysis time (F_(3, 200)_ = 3.63, *p* = 0.01). Post-hoc comparisons revealed that the mean percentage of covered analysis time of microstate D was lower (*p* = 0.02) in l-TLE as opposed to r-TLE patients (Fig. 6). We also found a significant main effect *Side* for the analysis of directional predominance (F_(1, 300)_ = 10.2, p < 0.01) that was higher in r-TLE compared to l-TLE (Fig. 7), with no interactions, ruling out a specific *Side* effect for selected microstates pairs of directional predominance.

Finally, we found no significant *Condition x Side* interactions across all microstates metrics (F_(1, 200)_ = 3.18, *p* = 0.07 for mean duration; F_(1, 200)_ = 2.83, *p* = 0.09 for occurrences per second; F_(1, 200)_ = 0.08, *p* = 0.77 for percentage of covered analysis time; and F_(1, 300)_ = 0.63, *p* = 0.43 for directional predominance), suggesting no specific effect of LEV therapy in microstates metrics differences between r-TLE and l-TLE.

## Discussion

In this study, we showed the effects of LEV as first ASM in a cohort of people with a new diagnosis of TLE through resting-state EEG microstate analysis. Our main findings can be summarized as follows: (i) LEV treatment induced a reduction of microstates B and D’s mean duration and (ii) microstates B and D’s occurrences per second and; (iii) LEV treatment increased the directional predominance of microstate A to C and microstate B to D.

### Microstate Metrics

Pharmaco-EEG studies in epilepsy have usually focused on assessing frequency modifications induced by old-generation ASMs, either visually or through quantitative analysis (Sannita et al. 1989; Wu and Xiao 1996, 1997; Höller et al. 2018). Only recently, previous works from our group showed that new-generation ASM therapy can induce a “normalization” of the EEG power spectrum and connectivity features in people with different types of epilepsy (Pellegrino et al. 2018; Lanzone et al. 2021; Ricci et al. 2021) and that such modifications are also predictive of good clinical outcome in TLE (Croce et al. 2021). Yet, to our best knowledge, microstate EEG analysis to evaluate the effects of ASMs in people with epilepsy has never been attempted before.

Microstate analysis is able to quantify long-range functional balances of networks, and such information has already been shown to provide prognostic information in patients after acute ischemic stroke (Zappasodi et al. 2017), probably because focal ischemic lesions directly impair the whole brain’s multi-scale systemic activity (Zappasodi et al. 2014).

Here, we found a reduction in microstate duration and occurrence in patients with TLE after 3 months of LEV therapy (Fig. 4). This is consistent with the literature since there is evidence that the metrics derived from microstate dynamics share rather similar neurophysiological implications (Zappasodi et al. 2017). The mean duration, named in several works as average lifespan, is considered to reveal the stability of the underlying neural networks, whereas the frequency of occurrence may reflect the propensity of a particular microstate and its neural generator to become activated.

Hence, a drop in microstate metrics can be considered as disentanglement and instability of the neural network generating the microstate topography, whereas an increase may be a sign of dysfunctional hyperactivity (Zappasodi et al. 2017). This is interesting since epilepsy has been increasingly recognized as a disorder of cortical networks (Englot et al. 2015; Assenza et al. 2020), and several studies have proposed that the EEG of people with epilepsy is characterized by abnormally synchronized networks and enhanced hyperconnectivity of the epileptogenic focus (Mormann et al. 2000; Iandolo et al. 2021; Ricci et al. 2021). Taken together, our results and previous works suggest a scenario in which LEV is able to disrupt a probably hyperfunctioning and abnormally active epileptic network. This notion is supported by the reduction in microstate metrics and the general good clinical response to LEV therapy in our cohort (11.1% with a < 50% reduction in seizure frequency; see Table 1).

Finally, we found that patients with r-TLE presented a higher mean percentage of covered analysis time for microstate D and a higher percentage of directional predominance compared to l-TLE. Microstate D significance has been functionally linked to the central executive and dorsal attention networks (Britz et al. 2010). Interestingly, a recent study using resting-state fMRI (Zhou et al. 2020) reported that network homogeneity in the right superior parietal lobule and right precuneus was significantly higher in patients with r-TLE than in patients in healthy controls, indicating alterations in the dorsal attention network in patients with r-TLE. Our own findings are consistent with this notion and suggest that in r-TLE, microstates metrics present increased general instability, which is reflected by an increase in the percentage of directional predominance, and a selective alteration in microstate D functioning, suggested by an increase in mean percentage of covered analysis time. Yet, we found no specific effect of LEV therapy in microstates metrics differences between r-TLE and l-TLE. To which extent microstate metrics among a larger cohort of r-TLE and t-TLE may differ after introducing different ASMs is a matter of future work.

### Microstate Templates and Directional Predominance

There is evidence that EEG microstates may be different in people with epilepsy as opposed to healthy controls. Indeed, a recent study showed that people with TLE presented alterations of microstate C parameters and that such features could differentiate epilepsy from healthy controls with an accuracy of 76.1% (Rajagopalan et al. 2018). Yet, there is a lack of knowledge about the modulation of EEG microstates induced by ASMs in people with epilepsy.

The EEG microstates display recurrent topographic distributions of the ongoing scalp potential fields and are proposed to represent the neurophysiological equivalent of the typical fMRI resting-state networks (Britz et al. 2010). In particular, microstate A has been linked to phonological and auditive processes, microstate B to visual activation, microstate C to the insula-cingulate salience network, and microstate D to the central executive resting-state network (Britz et al. 2010).

Our results showed a reduction in microstate B and D metrics and an increase in the directional predominance of microstate A to C and microstate B to D in newly diagnosed TLE patients after the initiation of LEV therapy.

Template D is mainly associated with the dorsal attention network (Britz et al. 2010). Several studies have demonstrated a selective impairment in dorsal attention networks in patients with TLE using resting-state fMRI (Zhang et al. 2009; Zhou et al. 2020). In particular, Zhou et al., reported an aberrant dorsal attention network homogeneity in patients with right TLE (Zhou et al. 2020). The reduction in microstate D observed in our cohort metrics may represent a neurophysiological biomarker of a reduction in the aberrant homogeneity of the dorsal attention network after the initiation of LEV. Yet, modifications in neuropsychological tests could not be verified in our cohort, given the retrospective nature of the study design. Changes in microstate metrics are reported to be influenced by certain behavioural states (Cantero et al. 1999) and impaired in patients with cognitive decline (Nishida et al. 2013). However, it should be mentioned that LEV is not associated with relevant cognitive side effects, as opposed to other ASMs (Gomer et al. 2007). Moreover, side-effects in our cohort of patients were rare and generally mild (18.5% of patients, see Table 1) and are unlikely to have influenced microstate parameters in our cohort. Crucially, microstate directional predominance unbalances with opposite alterations of B vs. D and A vs. C microstates as a modulation effect of LEV therapy underlines the relevance of network activity balances for brain functionality.

It is interesting to note that alterations in the dorsolateral prefrontal cortex (DLPFC), which is a key element of the salience network (microstate C) and central executive networks (microstate D), have been already described in TLE using quantitative MRI (Keller et al. 2009) and functional MRI (Qin et al. 2020). Indeed, Qin and colleagues found that TLE patients displayed impaired executive function, intrinsic alertness, and phasic alertness and orientation over time, and such cognitive decline was coupled with alterations DLPFC activity. They also described a slight improvement in cognitive functions over 3 years, suggesting that ASM therapy, better control of seizures, and shorter disease duration in TLE may have contributed to such modification in cognitive performance (Qin et al. 2020).

In this scenario, we may speculate that LEV may have induced a perturbation in the focal epileptic network, with reduced influence on the activity of resting-state global cortical networks. Definitely, our data support the notion that the focal alteration in the temporal lobe experienced by patients with TLE drives a global modulation of metrics and dynamics of microstates, with an increased representation of some states compared to others and higher transition probability which is triggered by the initiation of first ASM with LEV.

### Limitations and Future Directions

Our study has some limitations which should be reported. The first is the non-randomized, retrospective nature of the study design since our analysis was not directly designed for a clinical application and the results found on the group level in retrospection are not applicable in a clinical setting at single-subject level. However, our study showed that EEG microstate metrics extracted with low-density scalp EEG could unveil significant effects of ASM therapy and, once confirmed in larger cohorts, might be considered for possible future clinical applications with the aim of improving medical management and offering new potential biomarkers in people with epilepsy. In order to verify the stability and reproducibility of the microstates’ modifications induced by LEV, a further extension of our longitudinal assessment is warranted. Future longitudinal prospective studies performing periodic EEG recordings after the introduction of LEV at regular periods of time may effectively clarify this point.

All TLE patients in our cohort were drug naïve to other ASMs, and our results showed the modulation in microstate metrics induced by LEV therapy. However, the specificity of the effect of LEV on EEG microstates could not be completely pointed out at this time, given the lack of patients on other ASMs. Future studies evaluating different ASMs with multiple mechanisms of action may further clarify this point. We did not find an association between clinical variables (i.e., seizure-freedom after LEV, structural aetiology of TLE) and modulation in microstate metrics in our cohort. However, an attempt to find an association between different clinical variables (i.e., age, epilepsy duration, aetiology, location of the epileptic focus, type of ASM) and microstate metrics is beyond the scope of this work.

Changes in microstates are described in major depressive disorder (Murphy et al. 2020; He et al. 2021; Lei et al. 2022), and the presence of transient depressive symptoms as adverse events of LEV in two patients of our cohort may represent a possible confounder for the interpretability of our results. Yet, neither of these patients required anti-depressive treatment or LEV interruption, and such depressive symptoms were no longer described in further clinical follow-up.

Finally, it is important to emphasize that low-density EEG may present limitations due to its limited spatial resolution and incomplete coverage of mesial temporal lobe structures (Wennberg et al. 2011). In this regard, the use of more advanced neurophysiological techniques (i.e., high-density EEG) may offer new insights into the potential use of EEG microstates as neurophysiological biomarkers of ASMs activity and efficacy. Likewise, the reproducibility of our findings across other non-invasive protocols (i.e., high-density EEG, Magnetoencephalography) needs further investigation. Nonetheless, our study demonstrated that simple devices such as conventional low-density scalp EEG, which is low-cost and widely available in most neurophysiology units, may be exploited to analyze microstate EEG modifications induced by ASMs. Therefore, our approach can be applied even in centers that lack more advanced neurophysiological methods (i.e., magnetoencephalography or high-density EEG).

## Conclusions

This study shows that starting LEV treatment in newly diagnosed TLE patients induces a global modulation of resting-state EEG microstates metrics, which suggests an involvement of the focal epileptic network in the more global and widespread resting-state cortical networks. Microstate modifications induced by ASMs offer new insights into the identification of the neurophysiological effects of ASMs in the epileptic brain. The study of EEG microstates in people with epilepsy opens an interesting path to identify potential LEV activity biomarkers that may involve increased neuronal inhibition of the epileptic network.

## Data Availability

Data supporting our findings are available from the corresponding author, upon reasonable request.

## Abbreviations

ANOVA: Analysis of variance
ART: Aligned rank transform
ASMs: Anti-seizure medications
DLPFC: Dorsolateral prefrontal cortex
EEG: Electroencephalogram
fMRI: Functional magnetic resonance imaging
LEV: Levetiracetam
NSF: Non-seizure free
SF: Seizure-free
EEG_pre_: EEG performed before LEV initiation
EEG_post_: EEG performed after 3 months of LEV initiation
GD: Global dissimilarity
GFP: Global field power
KL: Krzanowski-Lai
MRI: Magnetic resonance imaging
TANOVA: Topographical analysis of variance
TLE: Temporal lobe epilepsy
